# A 3-day EGCG-supplementation reduces interstitial lactate concentration in skeletal muscle of overweight subjects

**DOI:** 10.1038/srep17896

**Published:** 2015-12-09

**Authors:** Jasper Most, Judith G P van Can, Jan-Willem van Dijk, Gijs H. Goossens, Johan Jocken, Jeannette J. Hospers, Igor Bendik, Ellen E. Blaak

**Affiliations:** 1 Department of Human Biology, NUTRIM School of Nutrition and Translational Research in Metabolism, Maastricht University Medical Centre+, Maastricht, The Netherlands; 2 Department of Human Movement Sciences, NUTRIM School of Nutrition and Translational Research in Metabolism, Maastricht University Medical Centre, Maastricht, The Netherlands; 3 DSM Nutritional Products, Basel, Switzerland

## Abstract

Green tea, particularly epigallocatechin-3-gallate (EGCG), may affect body weight and composition, possibly by enhancing fat oxidation. The aim of this double-blind, randomized placebo-controlled cross-over study was to investigate whether 3-day supplementation with EGCG (282mg/day) stimulates fat oxidation and lipolysis in 24 overweight subjects (age = 30 ± 2yrs, BMI = 27.7 ± 0.3 kg/m^2^). Energy expenditure, substrate metabolism and circulating metabolites were determined during fasting and postprandial conditions. After 6 h, a fat biopsy was collected to examine gene expression. In 12 subjects, skeletal muscle glycerol, glucose and lactate concentrations were determined using microdialysis. EGCG-supplementation did not alter energy expenditure and substrate oxidation compared to placebo. Although EGCG reduced postprandial circulating glycerol concentrations (P = 0.015), no difference in skeletal muscle lipolysis was observed. Fasting (P = 0.001) and postprandial (P = 0.003) skeletal muscle lactate concentrations were reduced after EGCG-supplementation compared to placebo, despite similar tissue blood flow. Adipose tissue leptin (P = 0.05) and FAT/CD36 expression (P = 0.08) were increased after EGCG compared to placebo. In conclusion, 3-day EGCG-supplementation decreased postprandial plasma glycerol concentrations, but had no significant effects on skeletal muscle lipolysis and whole-body fat oxidation in overweight individuals. Furthermore, EGCG decreased skeletal muscle lactate concentrations, which suggest a shift towards a more oxidative muscle phenotype.

The prevalence of obesity has become an epidemic problem during the last few decades^1^,^2^. Being overweight or obese is considered to be the most important risk factor for the onset of type 2 diabetes mellitus^3^.

In recent years there has been an increased interest in the health benefits of polyphenols in the prevention of obesity and type 2 diabetes mellitus. Green tea is rich in polyphenols, especially catechins. These catechins are comprised primarily of epigallocatechin -3- gallate (EGCG), epigallocatechin (EGC), and epicatechin (EC)^4^. Many of the beneficial health effects of green tea have been attributed to the most abundant catechin EGCG and were initially mainly related to their anti-oxidant activity^5^,^6^,^7^,^8^. More recently, interest has increased in the anti-obesity effect of green tea^9^. Consumption of green tea extracts (270 mg EGCG) in combination with caffeine supplementation (150 mg caffeine) has been shown to increase fat oxidation^10^,^11^ and energy expenditure^10^ in an acute manner in lean subjects, and to reduce body weight in overweight subjects^12^,^13^. However, studies are not consistent yet, which may relate to different doses of EGCG and caffeine, subjects’ BMI and genetic predisposition^11^,^14^,^15^,^16^,^17^. Preliminary data suggest that short-term EGCG supplementation may stimulate fat oxidation during postprandial conditions in overweight subjects^18^. Moreover, we recently showed that EGCG, in combination with resveratrol, increased fasting and postprandial energy expenditure, associated with an increased metabolic flexibility^19^, which may be associated with an increased insulin sensitivity^20^. Although supplementation of a green tea supplement for 24 hours in healthy men increased the Matsuda index^21^, reflecting a higher insulin sensitivity based on glucose and insulin concentrations after an oral glucose tolerance test^22^, most studies found no short-term effect on glucose homeostasis in humans^16^,^23^,^24^.

The underlying mechanisms behind the possible effect of green tea components on energy expenditure and lipid metabolism are not well studied in humans. One of the putative mechanisms is that EGCG may modulate energy expenditure by inhibiting catechol-o-methyltransferase (COMT)^10^,^11^, an enzyme involved in the degradation of norepinephrine^25^. As a consequence there is a prolonged stimulation of the adrenergic receptors, thereby increasing lipolysis and fat oxidation. Another mechanism may involve activation of sirtuins, especially sirtuin1 (SIRT1) and its transcriptional co-factor peroxisome proliferator-activated receptor-γ coactivator 1 alpha (PGC-1α), although few data of human studies are available^26^.

Watanabe *et al*.^27^ have shown that EGCG inhibited acetyl CoA-carboxylase (ACC) in 3T3-L1 cells suggesting that EGCG could alter the partitioning of lipids from storage towards oxidation. In addition, chronic feeding of green tea extract to mice has been shown to elevate skeletal muscle gene expression of factors involved in lipid transport and oxidation, such as FAT/CD36, medium-chain acyl-CoA dehydrogenase (MCAD) and uncoupling protein 3 (UCP3)^28^,^29^. Moreover, green tea extract reduced malonyl-CoA in skeletal muscle, which is an inhibitor of carnitine palmitoyl transferase (CPT), an enzyme involved in fatty acid transportation into the mitochondria^30^. The latter studies in mice, as well as our recent human study showing that combined EGCG and resveratrol supplementation affects flexibility of postprandial substrate oxidation without changes in systemic lipolysis^19^ may suggest that skeletal muscle is a major target tissue for the EGCG-induced metabolic effects. Nevertheless, whether the effects of EGCG and/or green tea extract in humans are mediated through direct effects on muscle metabolism, or secondary to a transient increase in free fatty acids (FFA) related to changes in adipose tissue metabolism or a regulatory action on gene expression remains to be established.

Therefore, we aimed to investigate whether a 3-day supplementation of 282 mg/day EGCG would increase fasting and postprandial fat oxidation, lipolysis (as indicated by circulating FFA and glycerol) and alter adipose tissue gene expression. Furthermore, in a subset of subjects (n = 12), local skeletal muscle metabolism was also assessed using microdialysis.

## Results

### Bioavailability

**Figure 1:**
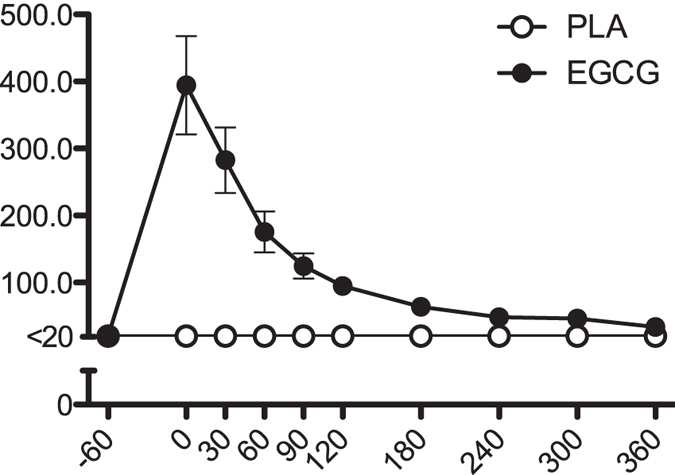
Time-course of plasma Epigallocatechin-gallate after oral intake of 300 mg/day or placebo. Results represent mean ± SEM; n = 24.

Plasma EGCG reached maximum concentration (394 ± 73 ng/mL (0.86 ± 0.16 μmol/L)) one hour after intake of EGCG (t = 0) and gradually declined below the detection limit (20 ng/mL) at the end of the meal-test (t = 360, Fig. 1). Relative bioavailability (AUC_plasma_/dose) was 19 ± 2%.

### Energy expenditure and substrate oxidation

**Figure 2:**
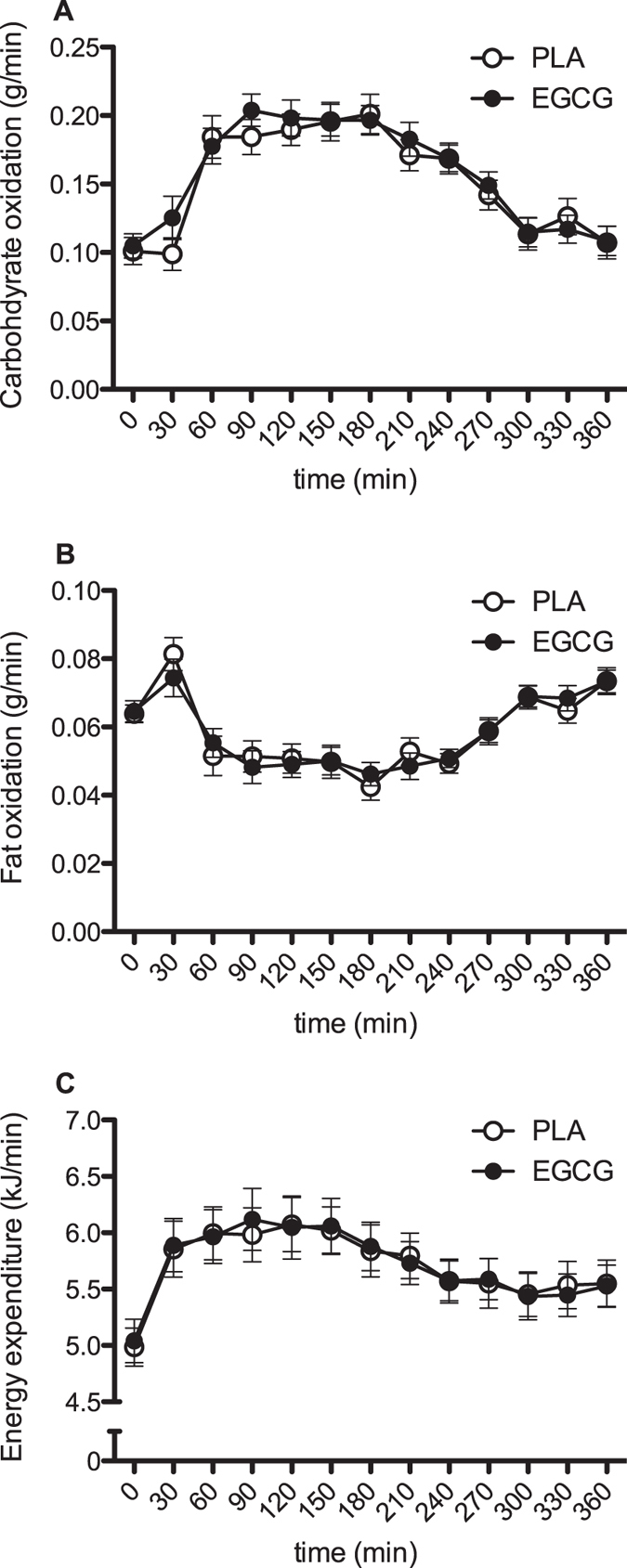
Time-course of substrate oxidation and energy expenditure after intake of 300 mg/day EGCG or placebo. Results represent mean ± SEM; n = 24. Carbohydrate oxidation (**A**), fat oxidation (**B**) and energy expenditure (**C**) before and after a mixed meal.

Supplementation of EGCG did not change fasting or postprandial fat and carbohydrate oxidation (Fig. 2A,B). Similarly, there were no differences in energy expenditure between conditions (Fig. 2C).

### Circulating metabolites

**Figure 3:**
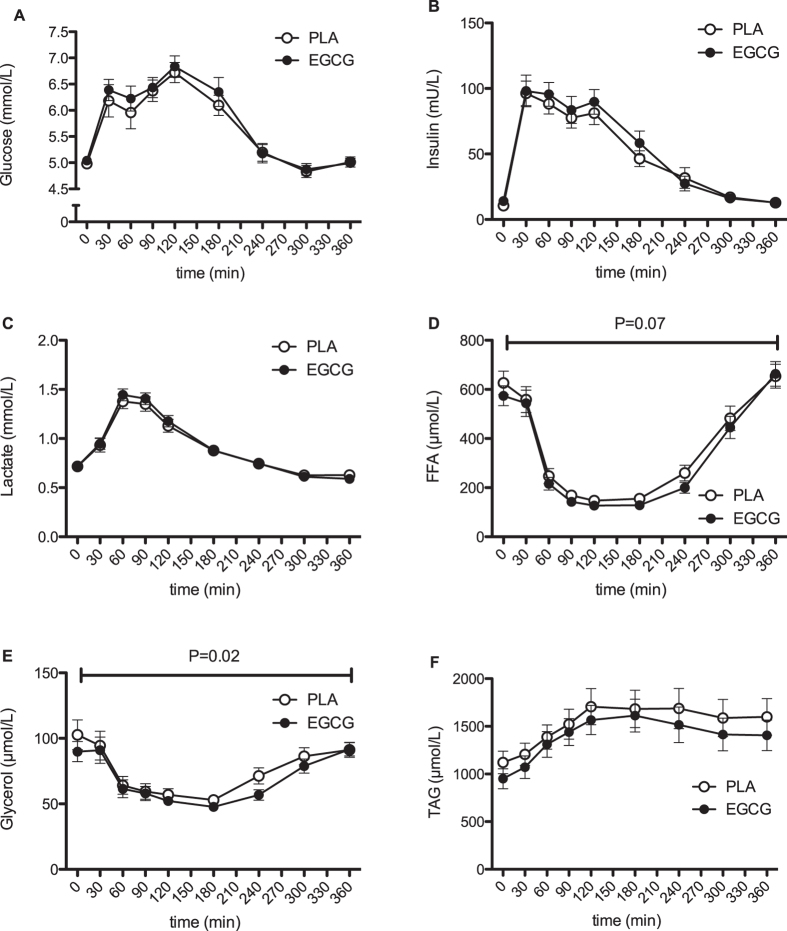
Time-course of systemic metabolite and hormone concentrations response after intake of 282 mg/day EGCG or placebo. Results represent mean ± SEM; n = 24. Plasma glucose (**A**), insulin (**B**), lactate (**C**), FFA (**D**), glycerol (**E**) and TAG (**F**) concentrations before and after a mixed meal. P: paired Student’s t-test for AUC-values.

EGCG supplementation showed no differences in plasma glucose, insulin and lactate concentration compared to placebo (Fig. 3A–C). Plasma free fatty acids (FFA) concentrations tended to be reduced after EGCG supplementation (AUC, P = 0.07, Fig. 3D). Furthermore, plasma glycerol concentrations were significantly reduced after EGCG supplementation as compared with placebo (AUC, P = 0.02, Fig. 3E), which was most pronounced in the late postprandial period (240–360 min). Plasma concentration responses to the meal, expressed as incremental area under the curve, were not affected by EGCG (p = N.S.). EGCG supplementation did not induce differences in plasma TAG concentrations, although fasting TAG concentrations tended to be reduced after EGCG (P = 0.07, Fig. 3F).

### Skeletal muscle lipolysis

**Figure 4:**
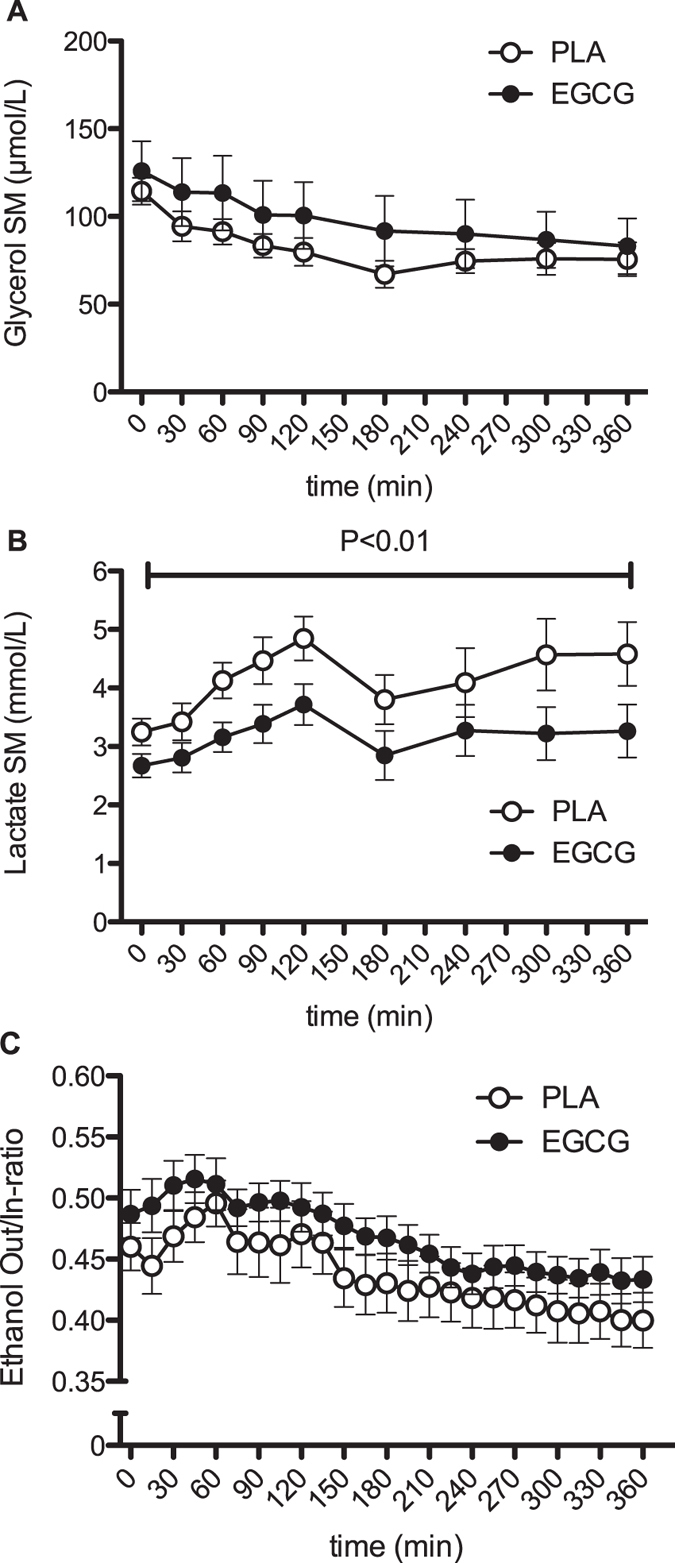
Time-course of interstitial metabolite concentrations in skeletal muscle after intake of 282 mg/day EGCG or placebo. Results represent mean ± SEM; n = 12. Skeletal muscle interstitial glycerol (**A**) and lactate (**B**) concentrations and ethanol Out/In-ratio (**C**) before and after a mixed meal. P: paired Student’s t-test for AUC-values.

Skeletal muscle interstitial glycerol, glucose concentrations, as well as ethanol in/out ratio were not different between EGCG supplementation and placebo (Fig. 4A,C, glucose data not shown). However, interstitial lactate was reduced with EGCG both for fasting levels (P<0.001) as well during the postprandial period (P = 0.003, Fig. 4B). Moreover, the postprandial increase of interstitial lactate concentrations tended to be attenuated by EGCG (iAUC, PLA: 15.2 ± 2.1 mmol/(L*360 min), EGCG: 9.8 ± 1.5 mmol/(L*360 min), P = 0.06).

### Adipose tissue gene and protein expression

The mRNA expression of CPT-1, ACC-1, adipose triglyceride lipase (ATGL) and hormone-sensitive lipase (HSL) were similar after supplementation with EGCG or placebo capsules. EGCG increased leptin (P = 0.05) and fatty acid translocase (FAT/CD36) (P = 0.08) mRNA expression compared to placebo (Table 1).

## Discussion

This study was designed to study the acute effects of EGCG supplementation (282 mg/day), the main catechin of green tea, on whole-body and skeletal muscle lipolysis and whole-body fat oxidation in overweight subjects. Supplementation of 282 mg/day EGCG for 3 days decreased circulating glycerol and tended to reduce FFA concentrations but did not alter local muscle lipolysis, substrate oxidation and energy expenditure compared to placebo. Skeletal muscle lactate concentrations were significantly reduced by EGCG compared to placebo, whilst skeletal muscle glucose concentrations were comparable, suggesting an ECCG-induced shift towards a more oxidative skeletal muscle phenotype. Bioavailability was in line with previously published data^31^.

It has been suggested that EGCG stimulates fat oxidation by augmenting sympathic nervous stimulation^10^,^32^, however human studies have shown conflicting results^19^,^33^. In the present study, no significant effect on postprandial fat oxidation and energy expenditure was found, which is consistent with findings of Lonac *et al*.^16^ (945 mg EGCG in 48 h) and Gregersen *et al*.^17^ (494–684 mg/day catechins and 150 mg caffeine) after 2 days EGCG, respectively acute EGCG-enriched catechin supplementation, in healthy adults. In contrast to our results, Thielecke *et al*.^24^ observed an increased fat oxidation in the first 2 h of the postprandial period following 3-day supplementation of EGCG. This may be explained by an extra dose of 150 mg EGCG that was ingested 1 h before meal intake, additional to the daily dose ingested in the morning. In addition, Dulloo *et al*.^10^ showed an increase in fat oxidation and energy expenditure following a single dose of catechins (375 mg catechins, 270 mg EGCG) + 150 mg caffeine in healthy young men. Since caffeine has been shown to independently stimulate energy expenditure in a dose-dependent manner, with doses as low as 100 mg showing effects^34^, synergistic effects of EGCG and caffeine could possibly explain the discrepancy between the study of Dulloo *et al*.^10^ and our present findings. Of note, in the study of Gregersen *et al*.^17^, lower doses of combined EGCG and caffeine (600 mg, respectively 150 mg, 6 small doses over 11 h) did not induce significant increases in fat oxidation and energy expenditure. The synergistic action of different supplements may explain the increased energy expenditure that we reported in overweight men and women after combined EGCG and RSV supplementation^19^.

Although there was no significant effect on postprandial fat oxidation in the present study, skeletal muscle lactate concentrations were reduced after EGCG supplementation both in the fasted state as well as during the postprandial period. These data might indicate a shift towards a less glycolytic and/or more oxidative muscle phenotype after EGCG. It is increasingly recognized that skeletal muscle lipolysis may play an important role in the regulation of mitochondrial function in skeletal muscle by activation of PPARs^35^,^36^. Since we found no significant differences in muscle lipolysis, this has not driven the shift in oxidative potential in muscle after EGCG supplementation. Also, we found no differences in adipose tissue lipase expression, and even reduced systemic FFA concentrations, indicating that the shift towards a more oxidative phenotype in skeletal muscle was not related to differences in the supply of exogenous fatty acids, but may possibly be due to a direct effect on mitochondrial function which has been reported earlier^26^,^37^. However, the reduction of interstitial lactate, indicative for a more oxidative phenotype, does not translate into significant alterations in substrate oxidation or glucose disposal. Still, even though the acute effects of EGCG on fat oxidation were not significantly different from placebo treatment, this does not rule out the possibility that green tea can have beneficial effects on fat oxidation^38^,^39^, body composition^12^,^13^,^38^,^40^ and insulin sensitivity^41^ over longer time periods. Therefore, the duration of supplementation, addition of caffeine or other polyphenols and combination with exercise are factors that have to be taken into account for future studies.

Systemic glycerol concentrations were significantly reduced in the postprandial period with EGCG, but not during fasting conditions. This decrease may indicate a slightly improved insulin-mediated suppression of adipose tissue lipolysis after EGCG treatment. Further studies are necessary to examine this mechanism or possible others, including glycerol clearance. Additionally, EGCG supplementation resulted in an upregulation of leptin mRNA expression in adipose tissue, whilst mRNA expression of the fatty acid transporter FAT/CD36 tended to be higher after EGCG supplementation compared to placebo. Similarly, we have previously found increased fasting concentrations of the adipose tissue derived satiety hormone leptin after short-term supplementation with EGCG and resveratrol^19^. In line, Josic *et al*.^42^ suggested that green tea might increase satiety although the data should be confirmed in a large clinical trial with overweight and obese subjects. Altogether, these data indicate slight effects on adipose tissue metabolism and function; more pronounced effects might require a longer period of supplementation and/or higher dosage of EGCG.

Lastly, the interaction between polyphenols and the gut microbiota may modulate the effect of EGCG supplementation^43^. Previous research suggests pre- and antibiotic properties of EGCG, which may influence peripheral metabolism through changes in microbiota composition and microbial products like short-chain fatty acids^44^. Moreover, it should be considered that microbial polyphenolic metabolites may have distinct effects on host metabolism^45^.

In conclusion, EGCG supplementation for 3 days decreased postprandial plasma glycerol concentrations, but had no significant effects on skeletal muscle lipolysis and whole-body fat oxidation in overweight individuals. Interestingly, EGCG decreased skeletal muscle lactate concentrations, suggesting a shift towards a more oxidative muscle phenotype. It can be speculated that the shift towards a more oxidative phenotype may be beneficial over a longer period in the prevention of obesity and related complications^20^.

## Methods

### Ethics Statement

The study was reviewed and approved by the Medical Ethical Committee of the Maastricht University Medical Centre^+^ and all subjects gave written informed consent before participation. All procedures were carried out in accordance with the approved guidelines.

### Subjects

Twenty-four overweight men (n = 9) and women (n = 15) with a low habitual caffeine intake (<300 mg/day) were recruited for this study. Sample size is calculated to detect a physiological relevant change in fat oxidation of 20% with a power of 90%, assuming a significance level of α = 0.05 and a drop-out of 20%, based on pilot data by Boschmann *et al*.^18^. Subjects‘ characteristics are presented in table 2. Subjects with type 2 diabetes and/or overt cardiovascular complications, and those using medication for digestive disorders were excluded from the participation. All subjects tolerated the EGCG capsules well and no adverse effects were observed on liver enzymes ALAT and ASAT.

### Study design

The effects of EGCG and placebo on postprandial fat oxidation were studied in a double blind, randomised cross-over design, with a washout-period of at least 7 days between both treatments. Subjects consumed the capsules during 2 days with breakfast and dinner (at both occasions 1 capsule of 141 mg). At day 3, subjects came to the university (Maastricht University Medical Centre+) for a test (test day). At this day the 2 capsules were ingested simultaneously, 1 h before the ingestion of a liquid mixed meal.

### Test product

The test product EGCG (Teavigo TG Lot: UT05080001) was provided by DSM Nutritional Products Ltd to Temmler Werke GmbH (Munchen, Germany). All capsules supplied by Temmler Werke GmbH were manufactured, tested and released according to Good Manufacturing Practice (GMP) guidelines. TEAVIGO TG™ contains >90% EGCG on a dry weight basis. The placebo capsules were filled with partially hydrolysed cellulose. The capsules were of identical appearance containing either 141 mg EGCG or placebo. 282 mg EGCG per day has been shown to be safe and well tolerated in single doses as well as repeated dosing^46^.

### Protocol Test day

All subjects were asked to refrain from drinking alcohol, smoking and doing strenuous exercise for a period of 24 h before the test day. Subjects came to the laboratory by car or bus in the morning after an overnight fast. At the beginning of the test day (day 3), a cannula was inserted into an antecubital vein for blood sampling. The EGCG or placebo capsules were consumed 1 h before metabolic testing and a fasting blood sample was drawn to determine baseline values of EGCG (t  =  −60 min). A liquid test meal was consumed 60 min after ingestion of EGCG/placebo capsules. Primary outcomes, energy expenditure and substrate utilization, were measured before and for 6 h after ingestion of the liquid test meal, using a ventilated hood system (Omnical, Maastricht University, The Netherlands)^47^. Gas analyses, which occurred every minute, are performed by dual paramagnetic O_2_ analysers and dual infrared CO_2_ analysers (type 1156, 1507, 1520; Servomex, Cowborough, Sussex, UK), similar to the analysis system described by Schoffelen *et al*.^48^. Blood samples were taken before ingestion of the liquid meal (t =  0 min) and for 6 h after meal ingestion at t =  30, 60, 90, 120, 180, 240, 300 and 360 min after EGCG/placebo ingestion to determine circulating metabolites and hormone concentrations.

The liquid meal had a total energy content equivalent of 40% of calculated 24 h resting energy expenditure based upon the formula of Harris and Benedict^49^. The energy content of the test meal was accounted for 49En% CHO, 35En% FAT and 16En% protein and was consumed within 20 minutes. A fat biopsy was taken at the end of the 6 h postprandial period in each condition (t  =  360 min). Blood samples and fat biopsy were snap frozen in liquid nitrogen and stored at −80 °C until analysis.

### Fat biopsy

A small amount (about 1 g) of abdominal subcutaneous adipose tissue was collected under local anesthesia using a needle biopsy technique and snap frozen in liquid nitrogen. Total RNA was isolated, using the total RNA stabilization and purification kit for human samples Qiagen (Qiagen, Hombrechtikon, Switzerland). Gene expression of HSL, ATGL, CPT-1, ACC-1, FAT/CD36 and leptin was measured by the Taqman multiplex method using the ABI 7900 quantitative real-time RT-PCR instrument (Applied Biosystems, Rotkreuz, Switzerland) as described by Heim^50^. All probe and primer sets were designed with the Primer Express program version 1.0 (Applied Biosystems) and initially tested to have comparable (>90%) efficiency in multiplex assays using 18S rRNA as an internal control. An overview of the primers and probes is listed in Supplementary Table S1.

### Microdialysis

In a subset of 12 subjects (6 men, 6 women, representative for the whole-study group with respect to subjects characteristics), the lipolytic effects of EGCG in skeletal muscle were determined by the microdialysis technique. On arrival, two microdialysis probes (CMA 60, CMA microdialysis AB, Stockholm, Sweden) were inserted in the medial portion of the *m. gastrocnemius* of both legs after anesthesia (xylocaine 2% without adrenalin, Astra Zeneca). Thereafter, 90 min was allowed for recovery of muscle from insertion trauma.

One probe was perfused with Ringer’s solution (147 mM sodium, 4 mM potassium, 2.25 mM calcium and 156 mM chloride, Baxter BV, Utrecht, The Netherlands) at a rate of 0.3 μL/min to obtain a near 100% recovery. Microdialysate was collected from these probes in 30 min fractions during the baseline period and during the early postprandial period (0–120 min) and at 60 min fractions during the last 4 h postprandially (120–360 min), to determine glycerol, glucose and lactate concentrations. Baseline concentrations of glycerol, lactate and glucose were determined by the average of three baseline samples. The second microdialysis probe was used for determining tissue blood flow using the ethanol dilution technique^51^,^52^. For this, the probes were perfused with Ringer’s solution supplemented with 50 mM ethanol, at a flow rate of 5 μL/min (Harvard microinfusion pump, Plato BV, Diemen, The Netherlands).

Ethanol concentrations were determined both in the ingoing and outgoing perfusion solvent to assess the ethanol inflow/outflow ratio as an indicator for local nutritive blood flow. Ethanol concentrations were determined at the same day, whereas microdialysate samples for measurement of glycerol, glucose and lactate concentrations were immediately frozen and stored at −80 °C until analysis.

### Biochemical analyses

At all time points, 8 mL blood was collected in pre-chilled tubes with 200 μL of 0.2 M EDTA (Sigma, Dorset, UK). After collection, blood samples were centrifuged immediately at 4 °C for 10 min at 1000 g and frozen at −80 °C until analysis. Additionally, 500 μL of the cell free plasma supernatant was combined with exactly 500 μL stabilization buffer at ambient temperature for the determination of EGCG concentration by HPLC. Plasma glucose (Uni Kit III, Roche, Basel, Switzerland), lactate, FFA (NEFA-C, Wako Chemicals, Neuss, Germany), TAG and free glycerol (148270, Roche Diagnostics, Indianapolis, IN, USA) concentrations were analyzed with a COBAS FARA semi-automatic analyzer (Roche). Insulin was analyzed by radioimmunoassay (Human Insulin RIA Kit, LINCO Research Inc, St. Charles, MO).

Glycerol, glucose and lactate concentrations from the microdialysates were measured by bioluminescence after enzymatic oxidation of L-Lactate^53^. Ethanol concentrations were measured spectrophotometrically using a standard enzymatic technique (R-Biopharm AG, Darmstadt, Germany).

### Calculations

Substrate oxidation was calculated from VO_2_ (L/min) and VCO_2_ (L/min) according to the equations of Frayn^54^. Nitrogen excretion was calculated based on the assumption that protein oxidation represents ~15% of total energy expenditure. Energy expenditure was calculated using the formula of Weir^55^. For each time point, the average of 20 minutes was used for calculation.

Carbohydrate oxidation (CHO) (g/min)  =  (4.55*VCO_2_)–(3.21*VO_2_)–(2.87*N)

FAT oxidation (g/min)  =  (1.67*VO_2_)–(1.67*VCO_2_)–(1.92*N)

N (g/min)  =  ((0.15* EE)/17)/6.25

### Statistics

All data are expressed as means ± SEM. The total response of parameters after ingestion EGCG or placebo was expressed as the total area under the curve (AUC) and calculated by the trapezoid method. Differences between placebo and EGCG were analyzed by means of student’s paired t-test. Normal distribution was tested by the Kolmogorov-Smirnov test. If parameters were not normally distributed they were ln transformed. Plasma lactate, TAG, glycerol and the genes CPT-1, ACC-1, HSL and leptin data were ln transformed. SPSS 15 for Windows was used to perform all calculation. The level of statistical significance was set at P ≤ 0.05.

## Additional Information

**How to cite this article**: Most, J. *et al*. A 3-day EGCG-supplementation reduces interstitial lactate concentration in skeletal muscle of overweight subjects. *Sci. Rep*. **5**, 17896; doi: 10.1038/srep17896 (2015).

## Supplementary Material

Supplementary Information

## Figures and Tables

**Table 1:** Normalized mRNA expression in adipose tissue after EGCG or placebo.

	EGCG	Placebo	P-value
CPT-1 mRNA	1,06·10^−5^ ± 1,0·10^−6^	9,3·10^−6^ ± 7,4·10^−7^	0.31
ATGL mRNA	3,51·10^−4^ ± 3,7·10^−5^	3,7·10^−4^ ± 3,0·10^−5^	0.12
HSL mRNA	5,38·10^−4^ ± 4,7·10^−5^	5,19·10^−4^ ± 4,1·10^−5^	0.73
FAT/CD36 mRNA	1,73·10^−3^ ± 1,2·10^−4^	1,52·10^−3^ ± 9,1·10^−5^	0.08
ACC-1 mRNA	2,16·10^−4^ ± 2,1·10^−5^	2,18·10^−4^ ± 2,6·10^−5^	0.81
Leptin mRNA	5,2·10^−4^ ± 5,9·10^−5^	4,3·10^−4^ ± 5,9·10^−5^	0.05

CPT-1, carnitine-palmitoyl-transferase-1; ATGL, adipose triglyceride lipase; HSL, hormone-sensitive lipase; FAT/CD36, fatty acid-translocase/cluster of differentiation; ACC-1, acetyl-Coenzyme-A-Carboxylase.

**Table 2:** Subjects’ characteristic’s.

	Mean ± SEM (N = 24, 9M/15F)
Age (years)	30 ± 2
BMI (kg/m^2^)	27.7 ± 0.3
Waist circumference (cm)	89 ± 1.7
Hip circumference (cm)	99 ± 1.1
WHR	0.90 ± 0.01
Fat mass (%)	28.8 ± 1.9
Fat free mass (%)	71.2 ± 1.9
Glucose (mmol/L)	5.20 ± 0.06
Insulin (μU/mL)	12.5 ± 0.7
HOMA-IR	2.9 ± 0.2
Systolic blood pressure (mmHg)	114 ± 2
Diastolic blood pressure (mmHg)	75 ± 1

BMI, Body mass index; WHR, Waist-to-hip ratio; HOMA-IR, Homeostatic assessment model for insulin resistance.
