# Supplementary material for: A 3-day EGCG-supplementation reduces interstitial lactate concentration in skeletal muscle of overweight subjects
Source: Sci Rep. 2015 Dec 9;5:17896. doi: 10.1038/srep17896 (PMC4673403; doi:10.1038/srep17896)
Supplement: Supplementary Information [file srep17896-s1.pdf]

Supplementary information

A 3-day EGCG-supplementation reduces interstitial lactate concentration in skeletal muscle of overweight subjects

Jasper Most[1], Judith GP van Can[1], Jan-Willem van Dijk[2], Gijs H Goossens[1], Johan Jocken[1], Jeannette J Hospers[3], Igor Bendik[3], Ellen E. Blaak[1].

[1] Department of Human Biology, NUTRIM School of Nutrition and Translational Research in Metabolism, Maastricht University Medical Centre+, Maastricht, The Netherlands.

[2] Department of Human Movement Sciences, NUTRIM School of Nutrition and Translational Research in Metabolism, Maastricht University Medical Centre, Maastricht, The Netherlands.

[3] DSM Nutritional Products, Basel, Switzerland.

Supplementary Table S1. Overview of primers and probes for RT-PCR of adipose tissue biopsies.

	forward	reverse	Probes
18S	CGGCTACCACATCCAAGGAA	GCTGGAATTACCGCGGCT	5'-VIC-TGCTGGCACCAGACTTGCCCTC-TAMRA-3
HSL	CTGCATAAGGGATGCTTCTATGG	CCTGTCTCGTTGCGTTTGTAGT	5'-FAM-CTGCCTGGGCTTCCAGTTCACGC-TAMRA-3
ATGL	TAGAGTGGCAGGTTGTCTGAAATG	CCCGTGTACTGTGGGCTCAT	5'-FAM-CACCATCCACGTAGCGCACCCC -TAMRA-3
CPT-1	CCATGTTGTACAGCTTCCAGACA	CACCGACTGTAGATACCTGTTCACA	5'-FAM-CTGCCTCGCCTGCCGGTCC-TAMRA-3
ACC-1	CAGCAGGCTGAACTTCACACA	CTGGAAGGCAGTATCCATTCATT	5'-FAM-CACGGATCCAGAGCACGGCACTC-TAMRA-3
Leptin	CCAAAACCCTCATCAAGACAATT	GAATGAAGTCCAAACCGGTGA	5'-FAM-CACGCAGTCAGTCTCCTCCAAACAGAAA-TAMRA-3

HSL, Hormone-sensitive lipase; ATGL, Adipose triglyceride lipase; CPT-1, Carnitine-Palmitoyl-transferase-1; ACC-1, Acetyl-Coenzyme-A-Carboxylase.
